# A Study to Evaluate the Effect of Cleaning Solutions on Shear Bond Strength of Resin Cement to Saliva-Contaminated Lithium Disilicate (LDS) Ceramic

**DOI:** 10.7759/cureus.44389

**Published:** 2023-08-30

**Authors:** Radhi RK, Divya Hegde, Juraise MC, Haseena Ummer, Nadeem Nazer, Jyothsna MK

**Affiliations:** 1 Prosthodontics, MES Dental College, Perinthalmanna, IND; 2 Prosthodontics, Bangalore Institute of Dental Sciences, Bangalore, IND; 3 Prosthodontics, Kannur Dental College, Anjarakandy, IND; 4 Prosthodontics, Educare Institute of Dental Sciences, Malappuram, IND

**Keywords:** ivoclean, resin composite, shear bond strength, sodium silicate solution, lithium disilicate ceramic, fixed dental prostheses

## Abstract

Background: One of the greatest benefits of contemporary restorative dentistry is the creation of fixed partial dentures. The retention and durability of the fixed partial dentures may be improved by using a variety of tooth preparation methods, surface treatments, and luting resins.

Aim: To measure the shear binding strength of resin cement to saliva-contaminated lithium disilicate (LDS) ceramic after being exposed to various cleaning treatments.

Methodology: Over 30 LDS samples were examined. In Group 1, a cleansing solution comprising 15% zirconium oxide (Ivoclean) was employed for a duration of 20 s. This was succeeded by a rinsing phase lasting 15 s utilizing deionized water, followed by a 15-s period for drying. Subsequently, a final stage of air drying was conducted over a span of 15 s. In contrast, Group 2 underwent a washing procedure of 20 s involving a cleansing solution containing 30% sodium silicate. This was then ensued by a rinsing interval of 15 s with deionized water, succeeded by an air-drying process spanning 15 s. After applying cleaning solutions to the surface and adhering the specimens to the composite blocks using resin cement Multilink Automix (Ivoclar Vivadent, Schaan, Liechtenstein), the shear bond strength was assessed.

Results: Using a 30% sodium silicate solution (Group 2), we were able to get the highest mean shear bond strength value after saliva-contaminated LDS. Group 1, 15% zirconium oxide (Ivoclean) had the weakest decontamination agents as measured by mean shear bond strengths. The shear bond strength values of the two groups were found to vary significantly between individuals using the independent sample *t*-test. LDs ceramic cleaned with a 30% sodium silicate solution had a higher shear bond strength than ceramic cleaned with Ivoclean (p<0.05).

Conclusion: The application of a 30% sodium silicate solution rendered the highest shear bond strength for saliva-contaminated LDS ceramic. In comparison, ceramic cleaned with Ivoclean exhibited notably lower shear bond strength values. The outcome of this investigation highlights the potential of diverse cleaning agents in influencing the adhesive qualities of resin cement, thereby contributing to the enhancement of fixed partial denture durability and efficacy.

## Introduction

One of the greatest benefits of contemporary restorative dentistry is the creation of fixed partial dentures. The retention and durability of fixed partial dentures may be improved by using a variety of tooth preparation methods, surface treatments, and luting resins [1]. Ceramics have exceptional optical qualities in addition to their chemical stability, which ensures their superior position among restorative materials. Ceramics have a low flexion performance, high rigidity, and pressure strength. Because of this, ceramic structures should be either supported by metal or more durable substructures that reduce the impacts of tensile strength on the surface, or they should be structurally fortified. Reinforced ceramics, which do not contain any metals, are gradually replacing this metal coping. As a result, esthetic restoration operations may benefit from the use of laminated ceramics reinforced with lithium disilicate (LDS) [2]. The use of LDS in dentistry has significantly increased. It is renowned for its mechanical qualities and adaptability in dental applications. It can be utilized to create different fixed restorations, including as single crowns and bridges with numerous units [1]. It has a further advantage over other materials in that its rate of fracture is incredibly low. It can endure a million-cycle ratio fatigue at 1,000 N of stresses [2].

The most up-to-date LDSs (IPS e.max Press/IPS e.max CAD, Ivoclar Vivadent, Amherst, NY) are monoblock restorations with a range of translucencies and opacities made possible by full press or milling production methods. Research shows that this material's low fracture rates are its greatest strength. The chemical composition and practical uses of LDS have led to its classification as a distinct kind of glass ceramic [2]. The components of IPS e.max LDS include quartz, lithium dioxide, phosphorus oxide, alumina, potassium oxide, and other elements. This durable glass ceramic was used in restorations made with either traditional hot-pressing methods or state-of-the-art computer-aided design and manufacturing milling processes. LDS in its pressable form is produced in ingots using a bulk-casting manufacturing method. Constant optimization of glass technologies (such as melting, cooling, simultaneous nucleation of two separate crystals, and development of crystals) prevents flaws (such as pores and pigments) from occurring in this continuous production process [2].

Bond strengths to high-strength ceramic materials may be further improved if the producer designs specialized surface patterns or employs nondestructive pretreatment processes [3]. The ability of hydrofluoric acid in varying quantities to cause morphological changes in reinforced ceramics by LDS is a unique property of this material. The etching process involves the controlled application of hydrofluoric acid at specific concentrations. Typically, concentrations ranging from 5% to 10% are utilized. The acid is meticulously applied to the ceramic surface for a defined duration, often around 20-60 s. This controlled exposure results in the creation of microretentive features on the ceramic surface, fostering enhanced adhesive bonding between the ceramic and resin cement. This etching mode, when conducted with precision, underscores the material's propensity for successful bonding in dental applications. The microstructural features of the material are to blame for this occurrence. Crystals of LDS, which are rather long and thin, make up the predominant crystalline phase. The lithium orthophosphate crystalline phase is the second one. Both crystalline phases are embedded in a glass matrix [4]. Microscopically, the pressable LDS material had a glassy matrix with needle-like LDS crystals making up around 70% of the microstructure. These crystals were between 3 and 5 mm in length. Glass ceramics were strengthened and were made to last longer than traditional dental ceramics by using LDS crystals [5]. Success rates for anterior and posterior LDS restorations ranged from 95.39% to 100%, according to recent research with a mean follow-up of 3 years [6]. In comparison, the glass ceramic inlays, onlays, and overlays demonstrated superior survival rates (97%) over a 5-year period [7]. Trims, onlays, delicate facades, facades, incomplete crowns, front and back crowns, three-unit anterior bridges and three-unit posterior bridge, telescope critical crowns, and embed superstructures are all proven to benefit from the use of pressable LDS [7-8]. Ceramic restorations failed if insufficient adhesion was achieved between the ceramic and tooth substrate. Clinical success was significantly aided by making the right choice in adhesive system. Hydrofluoric acid and silane coupling agents are indicated for the pretreatment of LDS to enhance bonding performance. Additionally, silane coupling agents serve a variety of purposes, including encouraging chemical connections between silica and a ceramic material's glass phase and the methacrylate groups in a resin cement by siloxane linkages. 

Hydrofluoric acid dissolved the surface to a depth of a few micrometers and damaged the ceramics' glassy phase, resulting in microporosities. The market has seen the introduction of a new class of bonding technologies (universal adhesives) recently. These adhesive solutions could attach to a wide variety of substrates, including resin composite, ceramics, zirconia, and metal alloys, and they were employed for both direct and indirect restorations. Some research has looked at how well a universal adhesive works for adhering resin to zirconia [9]. The wettability of the surface is essential for the ceramics to cling to one another regardless of the bonding technique utilized (chemical, mechanical interlocking, or a combination of the two). The durability of the bond between ceramic and resin depends on a number of factors, including the surface treatment of ceramics and the ability of the resin to moisten the bonding surfaces [9]. During the try-in procedure, contaminants like saliva, blood, or fitting indication remnants like silicone oil might contaminate the bonding surface of ceramic restorations. In addition to salivary proteins, enzyme molecules, bacteria, food waste, and salts, saliva is a combination of organic and inorganic components dissolved in water. Saliva may weaken the bond between restorations and the tooth substrate. However, throughout the try-in process, it was next to impossible to prevent. Therefore, prior to adhesive cementation, efforts were undertaken to remove any inorganic or organic impurities [10-12]. Consequently, sodium silicate is effective in removing contaminants from the repair surface and encouraging a strong connection [13].

Phosphoric acid gel is occasionally suggested for cleaning modern adhesive composite resins [14]. The bonding surface of restoration and its general strength may be preserved and impurities removed from universal restoration surfaces using the newly developed non-abrasive cleaning solution "Ivoclean" (Ivoclar Vivadent, Liechtenstein). However, there was insufficient evidence to back up the claims [15-17]. The shear bond strength of LDS ceramics to luting agents is affected by a number of factors.

While the organic pollutants like saliva and revealing media may be partially rinsed off with a water washing, the binding strength of resin cements is much lower than it would be on an uncontaminated ceramic surface [18]. This study aimed to determine which cleaning agents were most efficient in removing spit from the surface of LDS ceramics by measuring their effects on the shear bond strength of resin cement.

The hypothesis of the study was that using particular cleaning agents would have a substantial impact on the shear bond strength, illuminating their ability to improve or degrade the adhesive performance between LDS ceramics and resin cement.

## Materials and methods

Source of data

The study was conducted by the Department of Prosthodontics at the Bangalore Institute of Dental Sciences and Research Centre to compare the shear bond strength of resin cement to saliva-contaminated LDS ceramic after being exposed to various cleaning solutions. Thirty samples of LDS were tested in total. LDS specimen 10 mm in diameter and 3 mm in thickness. Composite block specimen 10 mm in diameter and 3 mm in thickness. A silicone mold was fabricated using elastomeric impression material (Photosil, Soft Putty, DPI), with the help of pre-made metal dies measuring 10 mm in diameter and 3 mm in thickness. The molten wax was poured into this mold. Using this wax pattern, the LDS (IPS e.max Press; Ivoclar Vivadent) specimen was made by the lost-wax hot-pressing technique. Lost wax casting is a casting process that uses a wax pattern to create a ceramic mold for creating a part or product design. All the lab samples were wet polished with 600-grit silicon carbide abrasive paper and conditioned as per the manufacturer's guidelines. Ultrasonic cleaner (Sidilu ultrasonic) was used on LDS samples in distilled water for 10 min. 

Fabrication of acrylic blocks to embed the lithium disilicate specimens

A cube-shaped wax pattern measuring 1 in. in height, length, and breadth was made using modeling wax. A prefabricated metal die was heated and embedded on one surface of the wax pattern. The metal die was removed once the wax was set. An equal quantity of additional silicone (Photosil, Soft Putty, DPI) was kneaded and the wax pattern was then placed into the kneaded impression material. Once the material was set, the wax pattern was retrieved. The obtained mold was lubricated with petroleum jelly and then the cold cure acrylic resin material was mixed and poured into the mold space. The resin blocks once set, were then removed and polished using an emery paper. Some 30 acrylic blocks were made using the elastomeric molds.

Fabrication of composite blocks

A silicone mold was fabricated using elastomeric impression material (Photosil, Soft Putty, DPI), with the help of pre-made metal dies measuring 10 mm in diameter and 3 mm in thickness. Into this mold, incremental layers of the composite were added and cured, until the specimen was obtained for the desired dimension. The obtained specimens were smoothened using diamond burs. After cleaning solutions were given to the specimens' surfaces and they were attached to composite blocks using resin cement, the shear bond strength was tested using a Universal Testing Machine (UTM) at the Indian Institute of Science (IISC) in Bangalore, India.

Surface treatment of lithium disilicate

The specimen has two sides/surfaces, one side used for marking the group and one side treated for bonding. Finally, cold-cure resin acrylic blocks were used to display each ceramic item. Before being placed in a sterile container, the samples were etched for 20 s in 5% hydrofluoric acid (IPS Ceramic Etching gel; Ivoclar Vivadent AG, Schaan, Liechtenstein), rinsed for 15 s in distilled water, and dried for 15 s before being put in their respective storage containers.

Grouping of specimen

A power analysis was established by G*power, version 3.0.1 (Franz Faul Universitat, Kiel, Germany). A sample size of 30 subjects (15 in each group) would yield 80% power to detect significant differences, with an effect size of 0.96 and a significance level of 0.05. A total of 30 specimens were created, including 15 specimens in each of two groups. 

Group I: Consisted of 15 specimens on which 15% zirconium oxide (Ivoclean) cleaning solution was treated after saliva contamination and cemented to composite blocks using Multilink Automix resin cement.

Group II: Consisted of 15 specimens on which 30% sodium silicate cleaning solution was treated after saliva contamination and cemented to composite blocks using Multilink Automix resin cement.

Saliva contamination

Healthy human saliva (clear and watery without an unpleasant odor) was obtained after the subject abstained from eating and drinking for 1.5 h. The healthy volunteer was asked to chew a piece of paraffin wax to stimulate salivation, and then to spit the saliva into a sterile container. The samples were submerged in human saliva for 1 min to check for contamination. After being submerged in deionized water for 15 s, the tainted samples were allowed to air dry for the same amount of time.

Cleaning methods

The following procedures were used to clean the samples after they had been contaminated with saliva. Group 1 used a zirconium oxide (Ivoclean) cleaning solution with 15% zirconium oxide for 20 s, followed by 15 s of rinsing with deionized water and 15 s of drying time, and finally 15 s of air drying. Group 2 washed for 20 s in a cleaning solution containing 30% sodium silicate, rinsed for 15 s in deionized water, and allowed to dry for 15 s in air.

Fabrication of composite blocks

A silicone mold was fabricated using elastomeric impression material (Photosil, Soft Putty, DPI, India), with the help of a pre-made metal die measuring 10 mm in diameter and 3 mm in thickness. Into this mold, incremental layers of the composite were added and cured, until the specimen was obtained for the desired dimension. The obtained specimens were smoothened using diamond burs.

Application of primer

The surface-treated LDS ceramic discs were primed with a single coat of universal primer (Monobond-S-Plus; Ivoclar Vivadent AG, Schaan, Liechenstein). After 60 s, a homogeneous coating of primer had formed and had been air-dried.

Resin cement bonding

Bonding a composite resin block to a ceramic surface under 1000 g of force required the employment of alignment equipment and the resin cement Multilink Automix (Ivoclar Vivadent, Schaan, Liechtenstein). Light activation for 20 s from two opposed sides, room temperature for 10 min, and storage in water at 370°C for 24 h followed by removal of excess cement.

Shear bond strength testing

The shear bond strength was measured using universal testing equipment after 24 h of storage. Employing a UTM, the shear bond strengths were tested at a cross-head speed of 1 mm/min until failure occurred.

Data were entered in the Excel spread sheet. Descriptive statistics like mean, standard deviation, and percentages were calculated. Inferential statistics like the independent sample *t*-test was used to find out the statistical difference between two groups for bond strength using SPSS (Statistical Package for Social Sciences) version 20 [IBM SPSS statistics (IBM Corp., Armonk, NY, released 2011)].

## Results

The results of the study are presented in Tables 1-2, which provide insights into the shear bond strength of resin cement to saliva-contaminated LDS ceramics, along with the effects of different decontamination agents. Table 1 displays the average shear bond strength of resin cement to saliva-contaminated lithium disilicate ceramics together with their standard deviations.

**Table 1:** Comparison of shear bond strength between the groups.

Groups	N	Minimum	Maximum	Mean	Standard deviation	
Group 1	15	16.05	19.12	17.80	0.85	
Group 2	15	20.34	24.73	21.83	1.24	

The groups are denoted as Group 1 and Group 2. For Group 1, consisting of 15 samples, the shear bond strength measurements range from a minimum of 16.05 MPa to a maximum of 19.12 MPa, with a mean shear bond strength of 17.80 MPa and a standard deviation of 0.85 MPa. In Group 2, also consisting of 15 samples, the shear bond strength measurements range from a minimum of 20.34 MPa to a maximum of 24.73 MPa, with a mean shear bond strength of 21.83 MPa and a standard deviation of 1.24 MPa. This table provides a clear overview of the shear bond strength characteristics of the two groups. Table 2 presents the comparison of shear bond strength between the groups using an independent sample *t*-test.

**Table 2:** Comparison of shear bond strength between the groups using independent sample t-test.

Variable	Mean difference	t Value	p Value
Group 1 vs Group 2	-4.02	-10.27	0.001

The variable of interest is the mean difference between Group 1 and Group 2. The calculated mean difference is -4.02, indicating that Group 2 exhibits a higher mean shear bond strength compared to Group 1. The t-value of -10.27 suggests a significant difference between the two groups. The p-value of 0.001 further confirms the statistical significance of the observed difference. This table highlights that Group 2 has a significantly higher shear bond strength than Group 1 when considering the effects of different decontamination agents.

Overall, these results suggest that the decontamination agent used in Group 2 led to a substantial increase in shear bond strength compared to Group 1. The findings emphasize the importance of decontamination agents in enhancing the adhesive properties of resin cement to saliva-contaminated LDS ceramics.

## Discussion

The success or failure of a treatment may be measured in part by how well the ceramic is bonded to the tooth structure utilizing the resin adhesive technique. But before final cementation, impurities on the inner surface of the bonding surfaces must be eliminated in order to ensure a strong connection [1]. The achievement of long-lasting bond strength is affected by the cleanliness of the bonding surfaces. A significant factor in the durability of restorations is the elimination of saliva impurities on ceramic inner surfaces prior to adhesion, which occurs during try-in procedures [15]. For successful bonding, the ceramic surface has to be treated chemically and physically to increase its wettability for the adhesive. In this experiment, LDS ceramic was exposed to hydrofluoric acid at a 5% concentration for 20 s to undergo a surface treatment. While silane, a bifunctional molecule, promotes ceramic-resin adhesion, reduces the contact angle, and increases the wettability of the ceramic surface, the acid dissolves the glassy phase in ceramic, increasing surface roughness and, consequently, a micromechanical interlocking occurs between the ceramic and resin cement. 

Lithium disilicate ceramic's glass matrix is attacked by hydrofluoric acid. Crystalline structure is revealed by removing the glass matrix selectively. This process roughened the ceramic's surface. Rough ceramic surfaces enhance surface energy, which in turn raises the likelihood of contamination and makes it more difficult to remove it [13]. Different solid substrates in the oral cavity, including enamel, dentine, and various restorative materials (glass ceramics, zirconia), have varying polarity and physical qualities that influence the adherence of the salivary proteins. Proteins, enzymes, glycoproteins, and other macromolecules from the saliva adsorb onto the restoration, creating a proteinaceous layer, during the try-in phase [15].

Several methods exist for cleaning up saliva contamination. This study used universal testing equipment to compare the shear bond strength of a resin composite to a clean and spit-contaminated LDS ceramic. In this experiment, we utilized Ivoclean and a 30% sodium silicate solution for cleaning [2]. Ivoclean is said to be a mixture of water, sodium hydroxide, and polyethylene glycol in which large-sized zirconia particles are suspended. During intraoral testing, salivary phospholipids attach to the ceramic surface, bringing with them any potential phosphate pollutants. The numerous zirconia particles in Ivoclean may be responsible for its decontaminating effects by selectively absorbing phosphate pollutants, hence cleaning the ceramic surface for efficient resin bonding. Using a shear bond strength test, Kim et al. [16] demonstrated that Ivoclean successfully removed impurities and increased bong strength values on zirconia surfaces.

Ivoclean was also proven to be successful in the disinfection of zirconia ceramics using saliva in research by Feitosa et al. [17]. Since sodium silicate solution is an alkaline cleaner, it was considered as a viable option. Because a liquid carrier medium is preferred for the cleaning composition of the invention, sodium silicate solution at a concentration of 30% was employed in this investigation. Cleaning using a sodium silicate solution did not substantially vary from cleaning with Ivoclean, however, it did result in the maximum shear bond strength on LDS ceramic [18].

Because of the treatment-induced topographical alterations, micro-mechanical retention and chemical binding with resin cements are improved. It was inevitable that the luting surface of the restoration would get contaminated with saliva, blood, or silicone indications during the clinical try-in process. Various factors through meticulous material selection, proper surface preparation, and adherence to recommended application protocols are essential to achieve consistent and durable ceramic-resin bonds in dental restorative procedures. However, the weakening of the bonds was mostly due to saliva contamination. So it was suggested that pre-cementation washing with cleaning solutions be done to eliminate the saliva contamination on the luting surface of the repair. If the ceramic's bonding surface is contaminated, the resin cement may not properly adhere to the ceramic.

The results of this investigation and statistical analysis indicate that when compared to Ivoclean, LDS ceramic that has been contaminated with saliva and treated with a 30% sodium silicate solution has strong shear bond strength to resin composite [19-20]. As a decontaminant, sodium silicate solution performed the best. Saliva from LDS ceramic was shown to be effectively decontaminated by sodium silicate. However, additional research and development is needed before it can be used effectively for cleaning in any environment.

The study has several limitations such as the study's design did not incorporate a long-term evaluation of the bonded specimens' durability and stability over extended periods of simulated oral function. The absence of fatigue testing or cyclic loading assessment prevents insights into the long-term performance of the bond under realistic conditions. Although a sample size calculation was performed, the study's sample size of 15 specimens in each group might be considered relatively small. Larger sample sizes could enhance the statistical power and reliability of the results, minimizing the risk of drawing conclusions from a limited data set. The study did not delve into the specific modes of failure that occurred during shear bond strength testing. Investigating the nature of failures could provide valuable insights into the actual bond integrity and the interplay between different factors. Additionally, the clinical implications of the observed differences in shear bond strength were not explored in terms of restoration longevity or patient outcomes. The absence of a control group in the study means that there was no comparison with unexposed, uncontaminated ceramic surfaces. This limits the ability to directly assess the impact of saliva contamination on bond strength and the effectiveness of the cleaning agents.

## Conclusions

The study's conclusions provide strong evidence that the sodium silicate solution is more effective than Ivoclean at decontaminating ceramics made of LDS. The resin composite's shear bond strength values, which are noticeably higher than average, are evidence of this increased effectiveness. Before stating this conclusion unequivocally, it is necessary to admit that a number of other relevant elements demand careful attention. Long-term performance, including factors like bond stability over time and under oral conditions, needs to be assessed to determine the sustained efficacy of the decontamination agents. The biocompatibility of the decontamination agents and their potential effects on adjacent tissues should be thoroughly evaluated to ensure patient safety and overall oral health. Further research could explore the specific mechanisms underlying the differences in decontamination efficacy and its direct correlation with shear bond strength enhancement. 
